# Complex Dynamics of Propagating Waves in a Two-Dimensional Neural Field

**DOI:** 10.3389/fncom.2019.00050

**Published:** 2019-07-30

**Authors:** Daniel Naoumenko, Pulin Gong

**Affiliations:** ^1^School of Physics, University of Sydney, Sydney, NSW, Australia; ^2^ARC Centre of Excellence for Integrative Brain Function, The University of Sydney, Sydney, NSW, Australia

**Keywords:** neural field, propagating waves, excitation, inhibition, spatiotemporal spectrum, traveling waves, cortical waves

## Abstract

Propagating waves with complex dynamics have been widely observed in neural population activity. To understand their formation mechanisms, we investigate a type of two-dimensional neural field model by systematically varying its recurrent excitatory and inhibitory inputs. We show that the neural field model exhibits a rich repertoire of dynamical activity states when the relevant strength of excitation and inhibition is increased, ranging from localized rotating and traveling waves to global waves. Particularly, near the transition between stable states of rotating and traveling waves, the model exhibits a bistable state; that is, both the rotating and the traveling waves can exist, and the inclusion of noise can induce spontaneous transitions between them. Furthermore, we demonstrate that when there are multiple propagating waves, they exhibit rich collective propagation dynamics with variable propagating speeds and trajectories. We use techniques from time series analysis such detrended fluctuation analysis to characterize the effect of the strength of excitation and inhibition on these collective dynamics, which range from purely random motion to motion with long-range spatiotemporal correlations. These results provide insights into the possible contribution of excitation and inhibition toward a range of previously observed spatiotemporal wave phenomena.

## 1. Introduction

Propagating waves have been observed at different neural levels within multiple recording techniques, including multi-electrode arrays (Freeman and Barrie, 2000; Rubino et al., 2006; Muller et al., 2014; Townsend et al., 2015; Zanos et al., 2015), voltage sensitive dye (VSD) imaging (Wu et al., 2008; Huang et al., 2010; Muller et al., 2014), electroencephalography (EEG), electrocorticography (ECoG), magnetoencephalography (MEG) and functional magnetic resonance imaging (fMRI) (Goldman et al., 1949; Ribary et al., 1991; Lee et al., 2005; Patten et al., 2012; Alexander et al., 2013). A growing body of evidence indicates that these waves are not just an epiphenomenon but have functional significance: in spontaneous activity, propagating waves have been shown to follow repeated temporal motifs instead of occurring randomly (Mohajerani et al., 2013; Townsend et al., 2015). They are postulated to facilitate information transfer across brain regions (Rubino et al., 2006; Sato et al., 2012) and carry out distributed dynamical computation (Gong and Van Leeuwen, 2009). In sensory and motor cortices, stimuli can elicit repeatable propagating waves and the properties of these waves can be linked to stimulus features (Prechtl et al., 1997; Xu et al., 2007; Wu et al., 2008; Sato et al., 2012; Muller et al., 2014). For instance, the phase and amplitude of traveling waves in the motor cortex and visual cortex correlate with reach target location (Rubino et al., 2006) and with saccade size (Zanos et al., 2015), respectively, and the propagation direction of moving waves in the visual cortex is sensitive to visual movement orientation (Townsend et al., 2017). Waves in this visual cortex are also implicated in perceptual phenomena like binocular rivalry (Lee et al., 2005), reinforcement of recent visual experience (Han et al., 2008) and visual hallucinations (Ermentrout and Cowan, 1979). More broadly, they have been observed during sleep with a possible role in memory consolidation (Botella-Soler et al., 2012; Muller et al., 2016). This body of evidence indicates that understanding the mechanisms behind the formation and modulation of propagating neural waves is essential for uncovering the principled dynamics of neural population activity and for understanding the working mechanisms of neural circuits (Muller et al., 2018; Townsend and Gong, 2018).

Propagating neural waves *in vivo* exhibit complex dynamics with variable propagation speeds and trajectories (Freeman and Barrie, 2000; Ferezou et al., 2007; Luczak et al., 2007; Han et al., 2008). It has been shown that such propagating neural waves with complex dynamics can emerge from spatially-extended, spiking neural networks (Keane and Gong, 2015), and the collective complex dynamics of these waves provides a mechanistic explanation for a range of irregular neural dynamics, including the variability of spike timing, slow firing rate fluctuations (Churchland et al., 2010), and correlated membrane potential fluctuations (DeWeese and Zador, 2006; Tan et al., 2016). The relative strength of excitation and inhibition is important for the emergence of such complex dynamics (Keane and Gong, 2015). In this study, we use a recently developed 2-D neural field model (Qi and Gong, 2015) to further investigate the dynamical impact of the neurophysiological mechanism of the relative E/I strength on wave dynamics. By varying the relative strength of excitation and inhibition, we find that a variety of propagating waves can emerge from the neural field, including localized rotating and traveling waves, splitting waves, and global waves. Based on their dynamical properties, we map out their presence on the E/I parameter space. Near the transition from the rotating state to the traveling state, there are co-existing propagating waves (i.e., plane and rotating waves); the noise-induced switching dynamics between these waves are systematically characterized. We further demonstrate that when there are multiple propagating neural waves, their interactions give rise to a range of pattern dynamics, including waves of purely random, Brownian motion and waves with great fluctuating dynamics that have long-range spatiotemporal correlations. These collective dynamics are systematically characterized using a range of methods including detrended fluctuation analysis and spectral analysis.

## 2. Materials and Methods

### 2.1. Neural Field Model

We consider a previously introduced two-dimensional neural field model with refractoriness (Qi and Gong, 2015) which is derived from a three-state spiking neural circuit model (Gong and Robinson, 2012). The refractoriness in the neural field model is in the form of non-linear negative feedback which is analogous to models that feature linear recovery variables (Pinto and Ermentrout, 2001), refractoriness (Meijer and Coombes, 2014) and synaptic depression (Kilpatrick and Bressloff, 2010; Bressloff and Kilpatrick, 2011). This particular model supports a wide variety of localized spatiotemporal patterns such as bumps and propagating waves. In this study, we investigate the effects of changing excitation and inhibition on the dynamics of individual pattern formation and propagation as well as their collective interactions.

**Figure 1:**
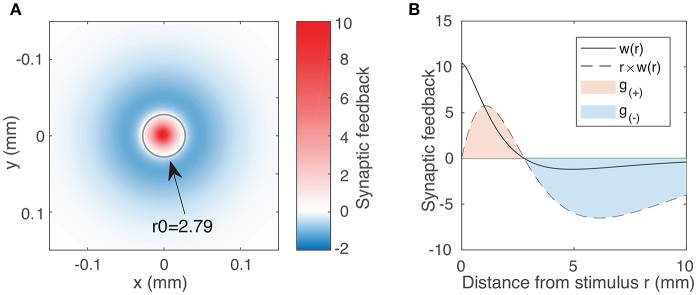
**(A)** Synaptic feedback to the rest of the neural field due to activity at the origin, as expressed by the weight function (Equation 3) using typical parameter values *W*_*E*_ = 144.4, *W*_*I*_ = 73.7, σ_*E*_ = 0.187 mm, σ_*I*_ = 0.324 mm. The labeled circle with radius 2.79 represents the transition between the excitatory region (*w*(*r*) > 0) and the inhibitory region (*w*(*r*) < 0). Although inhibitory feedback is weaker in magnitude than excitatory feedback, it affects a much larger region of the field. We account for this integrating over the excitatory and inhibitory region to obtain our excitation and inhibition parameters (Equation 5). **(B)** Weight function *w*(*r*) (solid line) as a function of distance *r* for the same parameter values as in **(A)**. The dotted line represents *r* × *w*(*r*), showing the synaptic feedback adjusted for the portion of the neural field receiving that feedback. The magnitudes of *g*_(±)_ are given by the areas of the red and blue shaded regions (Equation 5).

The model takes the form of a set of scalar integro-differential equations:

(1) $$\begin{array}{l} \tau \frac{\partial f}{\partial t}=-f+gH\left(u-\kappa \right)\\ \tau \frac{\partial g}{\partial t}=-gH\left(u-\kappa \right)+ph\\ \tau \frac{\partial h}{\partial t}=-ph+f, \end{array}$$

where, *f*(**r**, *t*), *g*(**r**, *t*), and *h*(**r**, *t*) are temporal coarse-grained variables that describe the probability that a neuron located at **r** ∈ **R**^2^ at time *t* is found in either a firing, refractory, or resting state, respectively. The parameter τ is the membrane time constant which we set to τ = 10ms, a typical value for cortical neurons (Koch, 2004). The parameter *p* expresses the refractoriness of the field: the rate at which a neuron transitions from a refractory state to a resting state. *H*(*x*) is the Heaviside step function: *H*(*x*) = 1 if *x* ≥ 0 and 0 otherwise. Here, it expresses the response of a neuron at **r** when its synaptic inputs *u*(**r**, *t*) exceed the threshold firing parameter κ:

(2) $$\begin{array}{r} u\left(\text{r},t\right)=\left(w*f\right)\left(\text{r},t\right)=\int_{{\text{R}}^{2}}w\left(|\text{r}-{\text{r}}^{\prime }\right)f\left({\text{r}}^{\prime },t\right)d{\text{r}}^{\prime }, \end{array}$$

where *w*(*r*) is a coupling function with short-range excitation and longer-range inhibition; as in Folias and Bressloff (2005), it is constructed from Bessel functions due to their analytic tractability:

(3) $$\begin{array}{l} \;w\left(r\right)=W_{E}w_{K}\left(r/\sigma_{E}\right)-W_{I}w_{K}\left(r/\sigma_{I}\right),\\ w_{K}\left(r\right)=\frac{2}{3\pi }\left[K_{0}\left(r\right)-K_{0}\left(2r\right)\right], \end{array}$$

where *K*_*i*_ is the modified Bessel function of the second kind; *W*_*E*_/*W*_*I*_ are the excitatory/inhibitory strength parameters, respectively; and σ_*E*_/σ_*I*_ are their corresponding spatial scales. Figure 1 shows the weight function vs. *r* for a set of fixed parameter values which produce a shape that approximately matches that of the original spiking model (Gong and Robinson, 2012). As in that model, spatial scales σ_*E*_, σ_*I*_ are chosen such that the size of the localized waves generated in the neural field matches that of waves observed in the cortex (Han et al., 2008).

For convenience, *t* is rescaled to the membrane time τ*t* → *t*. Further, by using the normalization condition *f* + *g* + *h* = 1, the neural field equations can be simplified to a pair of equations:

(4) $$\begin{array}{l} \frac{\partial f}{\partial t}=-f+\left(1-f-h\right)H\left(u-\kappa \right)\\ \frac{\partial h}{\partial t}=-ph+f, \end{array}$$

#### 2.1.1. Excitatory/Inhibitiory Parameters

In order to characterize the effect of varying excitation and inhibition on the activity within the neural field, we must first quantify the excitatory and inhibitory feedback as expressed by the weight function (Equation 3). Because this feedback varies with distance, we require a variable that reflects both the strength of the feedback and its spatial extent. A natural place to start is to consider the 2D integral of the weight function. For the limits, we inspect the weight function, denoted by the solid line in Figure 1B. We note that feedback is positive (excitatory) up to a certain distance we label *r*_0_ and negative (inhibitory) beyond that. We thus introduce the following variables to quantify the excitatory and inhibitory feedback by integrating over two separate regions in space **r**:

(5) $$\begin{array}{r} g_{\left(+\right)}=\underset{|\text{r}|<r_{0}}{\iint }w\left(r\right)d\text{r}\\ g_{\left(-\right)}=\underset{|\text{r}|>r_{0}}{\iint }w\left(r\right)d\text{r} \end{array}$$

The dotted line in Figure 1B represents the value of *r* × *w*(*r*). Hence, the area of the shaded regions represents the total excitatory and inhibitory inputs to the rest of the field. These quantities vary non-linearly and in a coupled way with respect to all four weight function parameters (the coupling strengths *W*_*E*_, *W*_*I*_ and the spatial scales σ_*E*_, σ_*I*_). We proceed by using *g*_(±)_ as our excitatory/inhibitory parameters.

In order to numerically characterize the dynamical effects of excitatory and inhibitory feedback on the neural field, we need to properly sample the *g*_(±)_ parameter space. Since the parameters *g*_(±)_ vary non-linearly with respect to the weight function parameters, it is not possible to evenly sample the parameter space with a scheme of linear variation. Furthermore, a given pair of values *g*_(+)_ and *g*_(−)_ does not correspond to a unique weight function; different combinations of *W*_*E*_, *W*_*I*_, σ_*E*_, and σ_*I*_ can produce similar but not identical weight functions with the same values of *g*_(+)_ and *g*_(−)_. In order to eliminate the sampling biases that can arise from a particular scheme of variation, we first use a Monte Carlo approach to sample the *g*_(±)_ parameter space by generating random values of *W*_*E*_, *W*_*I*_, σ_*E*_ and σ_*I*_. We use this to explore general trends and validate the use of *g*_(±)_ as a measure of excitation/inhibition in the model. For more demanding calculations and simulations that would be unfeasible to do for all of the *g*_(±)_ pairs generated using the Monte Carlo approach, we instead use a smaller set of parameters, found by partitioning the *g*_(±)_ parameter space into an evenly-spaced grid. For each pair of *g*_(±)_ values, we then find corresponding values of *W*_*E*_, *W*_*I*_, σ_*E*_, σ_*I*_ by applying a non-linear optimization algorithm to Equation (5). To do so, we introduce an objective function $f_{obj}=g_{\left(+\right)}^{2}+g_{\left(-\right)}^{2}$ which can then be minimized using one of many widely-available non-linear numerical solvers. We use the algorithm outlined in Byrd et al. (2000), although the results do not depend on the choice of algorithm.

For the Monte Carlo approach, we generate random weight function parameters by uniformly sampling each parameter (*W*_*E*_, *W*_*I*_, σ_*E*_ and σ_*I*_) in the interval (0, 150). For each combination, we evaluate Equation (3) and discard combinations which do not result in a profile consisting of short-range excitation and long-range inhibition. To explore the dynamics of the field under varying excitation and inhibition, we first consider the effects on the simplest form of spatiotemporal activity: localized, radially symmetric bumps.

## 3. Results

### 3.1. Bump Solutions

#### 3.1.1. Dependence of Radius on Excitation and Inhibition

Localized radially symmetric bumps are solutions to the neural field equations which have a constant, uniform value (*f, h*) within their circular boundaries. Their simple form results in a closed expression that is analytically tractable. For a bump of radius ρ centered at the origin, these solutions have the following form (Qi and Gong, 2015):

(6) $$\begin{array}{l} f(r)=\{\begin{array}{cc} \frac{p}{1+2p} & \text{for }r<\rho \\ 0 & \text{for }r>\rho \end{array},\\ h(r)=\{\begin{array}{cc} \frac{1}{1+2p} & \text{for }r<\rho \\ 0 & \text{for }r>\rho \end{array}. \end{array}$$

At the boundary of the bump, the sum of synaptic input *u*(**r**, *t*) (Equation 2) must approach κ. This leads to the following existence condition for all bumps:

(7) $$\begin{array}{r} \frac{p}{1+2p}\left[\int_{0}^{2\pi }\int_{0}^{\rho }w\left(\left|\text{r}-{\text{r}}^{\prime }\right|\right)r^{\prime }dr^{\prime }d\theta^{\prime }-2\kappa \right]-\kappa =0 \end{array}$$

**Figure 2:**
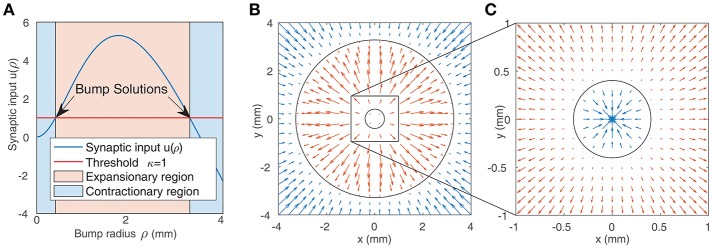
**(A)** Synaptic input at the boundary of a bump as a function of bump radius (as given by Equation 7). Solutions are indicated by the arrows. The blue shaded region represents bumps whose synaptic input at the boundary is inhibitory, leading to contraction of the bump, while the red region represents bumps whose synaptic input at the boundary is positive, leading to expansion. **(B,C)** Representation of the boundaries of two bump solutions from **(A)** in the neural field (black circles). The arrows show the value of synaptic input at the boundary of bumps with different radii. Positive values (red arrows) result in expansion, while negative values (blue arrows) result in contraction. This implies that the smaller bump in **(C)** is unstable while the larger bump in **(B,C)** is stable.

We solve this equation numerically to find the bump radius ρ for a given weight function. Figure 2A shows the synaptic input at the bump boundary *u* as a function of the bump radius ρ, with the red line showing the value of the firing threshold κ. Bump solutions occur where the curve intersects the line. Due to the concave profile of *u*(ρ), there can be at most two solutions. The gradient of *u*(ρ) at the solutions implies that the smaller bump is unstable while the larger bump is stable: if the synaptic input at the boundary is negative, this results in a decrease in activity and thus a reduction in the bump radius; if the synaptic input is positive, this results in an increase in the bump radius. The positive gradient of *u*(ρ) at the smaller bump indicates that a small perturbation will result in either extinction or expansion to the larger bump solution, while the negative gradient of *u*(ρ) at the larger bump solution suggests that small perturbations will tend to return the bump to its original solution. This is in agreement with direct numerical simulations and is presented in Figures 2B,C.

**Figure 3:**
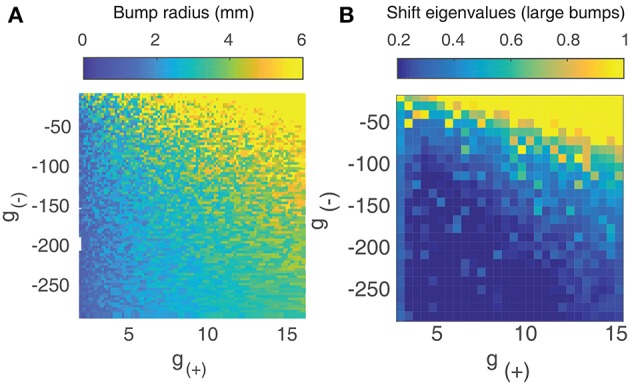
**(A)** Radius of large bump solutions as a function of *g*_(+)_ and *g*_(−)_. We fix *p* = 0.42 as this was approximately the critical value identified by Qi and Gong (2015) at which bump solutions were found to become unstable for the weight function parameters used in Figure 1. Increasing excitation or decreasing inhibition results in larger bumps. **(B)** Eigenvalues of large bump solutions with respect to shift perturbations are positive for all values of *g*_(+)_ and *g*_(−)_, becoming larger as excitation is increased or inhibition is lowered.

Since the smaller bump solutions are unstable, we proceed by focusing on the larger branch of solutions. As shown in Figure 3A, the radius of the larger bumps depends on the parameters *g*_(±)_. Decreasing inhibition *g*_(−)_ or increasing excitation *g*_(+)_ results in an increase in the radius of the bumps. As experimentally observed waves in the cortex have a radius of around 2–4 mm (Han et al., 2008), we only explore the region of the *g*_±_ parameter space that corresponds to patterns of this size.

#### 3.1.2. Dependence of Stability on Excitation and Inhibition

To quantify the influence that the level of excitation/inhibition has on these solutions, we perform a linearly stability analysis on the larger branch of solutions. We consider linear shift perturbations, as these correspond to the transition from stationary bumps to traveling waves. We follow the method of Qi and Gong (2015) to numerically evaluate the largest eigenvalue of the perturbation, as shown in Figure 3B. We find that eigenvalues are positive for all values of *g*_(±)_, increasing significantly as excitation is increased or inhibition is weakened. This indicates that for *p* = 0.42, radial bumps will always be unstable even if the level of excitation and inhibition is changed. Although Qi and Gong (2015) showed that lower values of *p* do result in stable bump solutions with negative eigenvalues for shift perturbations, we do not consider them as we wish to explore the collective dynamics of interacting traveling waves, which are only seen at higher values of *p*.

### 3.2. Propagating Waves

We now study the behavior of more complex patterns such as traveling waves under varying excitation and inhibition. Though we are interested in the behavior of waves in a two-dimensional neural field, we begin by considering the one-dimensional case in order to develop insights that are applicable to the 2D case and to validate subsequent observations made using direct numerical simulations. By considering the mathematical form of traveling waves we can quantify and semi-analytically characterize the variation we observe when varying the level of excitation and inhibition.

#### 3.2.1. Traveling Waves in One-Dimension

When considering the 1D case, we can describe a traveling wave with just two parameters: it's length *L* and speed *c*. From Qi and Gong (2015), the equations of a traveling wave whose front lies at the origin and is traveling in the positive *x* direction are:

(8) $$\begin{array}{l} F(x)\text{ =}\{\begin{array}{ll} 0, & \text{if }x>0\\ \frac{p}{1+2p}\left\{1-\exp(\alpha x/c)[\cos(\beta x/c)-A\sin(\beta x/c)]\right\}, & \text{if }-L\leq x\leq 0\\ F(-L)\exp[(x+L)/c], & \text{if }x<-L \end{array}\\ H(x)\text{ =}\{\begin{array}{ll} 0, & \text{if }x>0\\ \frac{1}{1+2p}\left\{1-\exp(\alpha x/c)[\cos(\beta x/c)-B\sin(\beta x/c)]\right\}, & \text{if }-L\leq x\leq 0\\ H(-L)+\frac{F(-L)}{1-p}\exp[p(x+L)/c]-\frac{F(-L)}{1-p}\exp[(x+L)/c], & \text{if }x<-L, \end{array} \end{array}$$

where $\alpha =\left(2+p\right)/2,\beta =\sqrt{p\left(4-p\right)}/2,A=\left(2-\alpha +1/p\right)/\beta$ and *B* = {α − 2 − *p*[(α − 2)^2^ + β^2^]}/β. Since the synaptic input *u*(*x*) at the two boundary points *x* ∈ {0, −*L*} must equal the threshold κ, we can find solutions by numerically solving the following equations over *c* and *L*:

(9) $$\begin{array}{l} \;u(0)=\int_{-\infty }^{0}w(x)F(x)dx=\kappa \\ u(-L)=\int_{-\infty }^{0}w(x+L)F(x)dx=\kappa \end{array}$$

**Figure 4:**
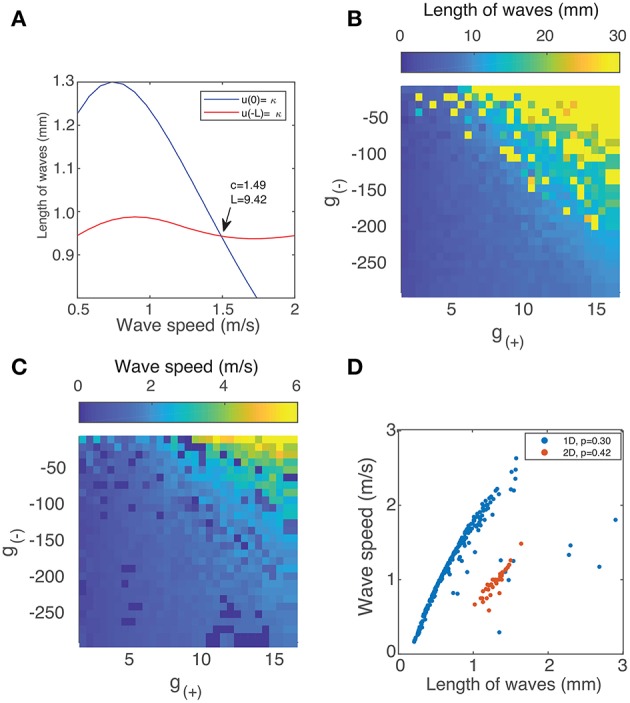
**(A)** Method of finding the speed and length of waves in 1 dimension by simultaneously solving Equation (9). **(B)** Length of traveling waves in 1 dimension vs. the parameters *g*_(±)_ using *p* = 0.3. **(C)** Speed of traveling waves in 1 dimension vs. *g*_(±)_ using *p* = 0.3. **(D)** Wave speed vs. wave velocity in both 1D (blue) with *p* = 0.3 and 2D with *p* = 0.42 (red). The method used to obtain the results for 2D waves is outlined in section 3.3.

The two curves described by Equation (9) are shown in Figure 4A. They intersect at a single point, which we verify to be a solution using direct numerical simulation. By simultaneously solving both equations numerically for *c* and *L*, we can find traveling wave solutions for any value of *g*_±_. To do so, we introduce a single objective function formed by summing the squares of Equation (9):

$$f_{obj}={\left(u(0)-\kappa \right)}^{2}+{\left(u(-L)-\kappa \right)}^{2})$$

Using a multi-parameter optimization algorithm (Byrd et al., 2000), we find values of the wave speed *c* and length *L* that minimize *f*_*obj*_. Figures 4B,C show the length and speed of traveling waves in 1 dimension as a function *g*_(+)_ and *g*_(−)_. We find that there exists a strong relationship between the strength of excitation/inhibition and the behavior of traveling waves: the speed and length of traveling waves increases as excitation increases or inhibition decreases. Figure 4D shows how the speed of the waves is correlated with their length. Most of the solutions lie on a curve, with a strong positive correlation between the two parameters. All the (*c, L*) solutions also lie on one side of this curve, which suggest that there is a frontier bounding possible (*c, L*) values and that there may be more than one solution for a given value of *g*_(±)_. This is also observed in direct numeric simulations, where there can be several traveling wave solutions for a given value of *g*_(±)_, although one solution will have greater velocity and length than all others.

##### 3.2.1.1. Two-dimensional case

We now consider the case of propagating waves in a two-dimensional neural field. For this, we must use direct numerical simulation as the inclusion of refractoriness in the neural field model means there are no simple methods for finding a closed-form traveling wave solution which would be necessary for an analytical approach. Direct simulation, however, still requires an appropriate initial condition for the neural field in order to simulate a wave, as they do not always emerge spontaneously. As with the one-dimensional case, the form of these waves (their shape and propagation speed) varies with the parameter values. Hence, we cannot use the same initial state for every simulation, particularly as for some parameter values, the neural field can support other types of activity such as rotating waves. In these cases, it is important that the initial state be very close to the traveling wave solution.

**Figure 5:**
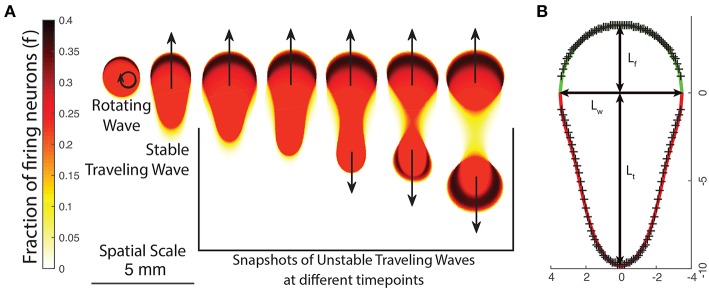
**(A)** The changing appearance of patterns as the neural field parameters are varied, either by changing *p* or the excitation/inhibition parameters *g*_±_. As patterns become more unstable, they transition from rotating waves to traveling waves and unstable waves which undergo periodic division. **(B)** Empirically fitting a function curve to the wave front (green line) and tail (red line) for a traveling wave. Crosses show boundary points obtained from numeric simulation.

In order to generate suitable initial states, we develop a method to numerically find an approximate traveling wave solution for any given set of parameter values. We note that we can apply Equation (9) along the direction of propagation to find the value of (*f, h*) inside the wave boundary, but we must know both the speed of the wave and its shape (boundary) beforehand. We proceed by trying to find an empirical equation for the general boundary of a traveling wave. As a start, we use the shapes of waves that emerge spontaneously from an initial state consisting of a deformed bump solution. Varying *p* leads to various types of waves; these are presented in Figure 5A.

We first note that traveling waves have a characteristic teardrop or comet shape with a distinct front and tail which we separate by a line through their widest point. We thus propose the following general parametric form for the boundary of a traveling wave centered at the origin in polar coordinates, with ρ_*trav*_(θ) being the boundary of the traveling wave and $\theta \in \left[-\frac{\pi }{2},\frac{3\pi }{2}\right)$:

(10) $$\rho_{trav}(\theta )=\{\begin{array}{cc} \rho_{front}(\theta ) & \text{if }\theta \leq \pi /2\\ \rho_{tail}(\theta ) & \text{if }\theta >\pi /2 \end{array}$$

This gives three global parameters: the front length *L*_*f*_, tail length *L*_*t*_ and the width *L*_*w*_. We find that the front is well described by the equation of an ellipse:

$$\rho_{front}\left(\theta \right)=L_{f}L_{w}/\sqrt{L_{w}^{2}\cos^{2}\theta +L_{f}^{2}\sin^{2}\theta }$$

The tail, however, has a more complex shape. From testing various families of equations, we find that the following equation is a good fit to the boundary obtained from numeric simulations and only requires one additional parameter:

(11) $$\begin{array}{r} \rho_{tail}\left(\theta \right)=L_{w}-L_{a}\left[1-1/\left(1-L_{b}\cos^{2}\theta \right)\right]. \end{array}$$

*L*_*a*_ and *L*_*b*_ are parameters that describe the curvature of the tail and are related to the tail length *L*_*t*_ by: $L_{t}=L_{w}+L_{a}L_{b}/\left(1-L_{b}^{2}\right)$ and 0 < *L*_*b*_ < 1. Using Equations (8) and (10), we have a total of five parameters that describe a traveling wave uniquely: *c, L*_*f*_, *L*_*w*_, *L*_*a*_, *L*_*b*_. One such wave is shown in Figure 5B. In order to find traveling wave solutions for a given value of *g*_±_, we must find parameters such that the synaptic input along the boundary of the wave equals the threshold value κ:

(12) $$\begin{array}{r} u\left(\text{r}\left(\rho_{trav}\left(\theta \right),\theta \right)\right)=\int_{{\text{R}}^{2}}w\left(\left|\text{r}-{\text{r}}^{\prime }\right|\right)F\left({\text{r}}^{\prime }\right)d{\text{r}}^{\prime }=\kappa , \end{array}$$

where *F* is a traveling wave moving in the positive *x* direction:

$$\begin{array}{l} F\left(\text{r}\right)=F\left(\sigma_{+}\left(y\right)\right){|}_{\left(L=\sigma_{+}\left(y\right)-\sigma_{-}\left(y\right)\right)}, \end{array}$$

and σ_±_(*y*) is the *x*-coordinate of the front (+) or tail (−) of the wave for a given *y*-coordinate.

Unlike the one-dimensional case where we only had two discrete boundary conditions, here we have a continuous boundary. In order to solve Equation (12) numerically, we can discretize the boundary $\theta \in \left[-\frac{\pi }{2},\frac{3\pi }{2}\right)$ over *n* points and minimize the following objective function over a finite number of points θ_*i*_ on the boundary:

(13) $$\begin{array}{r} f_{obj}\left(c,L_{f},L_{w},L_{a},L_{b}\right)=\overset{n}{\underset{i=1}{\sum }}{\left[u\left(\text{r}\left(\rho_{trav},\theta_{i}\right)\right)-\kappa \right]}^{2}. \end{array}$$

This function is highly nonlinear with many local minima, so we apply a pattern search algorithm as outlined in Audet and Dennis (2002) in order to find values of *c, L*_*f*_, *L*_*w*_, *L*_*a*_, *L*_*b*_ that minimize Equation (14). What we observe, however, is that this does not give us a unique solution: it is possible to find several similar but distinct traveling waves that minimize Equation (14). We verify the validity of these solutions using direct numerical simulation. We discretize spatially and temporally and apply a numerical scheme (Fourth-order Runge-Kutta as in Butcher, 2007) to the integro-differential equations (Equation 4). We use a spatial resolution of 0.1 mm with a grid size of 601 × 601 and a temporal resolution of 0.1 ms. We set κ to 1 and *p* to 0.42. In order to explore the parameter space, we linearly sample *g*_±_ pairs as outlined earlier in section 2.1. We simulate the neural field and then plot the final length and speed of the traveling waves on Figure 4D, alongside that of the one-dimensional waves. As with 1D waves, we observe that 2D traveling waves have a positive correlation between wave speed and velocity, with a similar “frontier” bounding possible values. When the wave velocity is low, we find that traveling waves tend to transition to rotating waves; when wave velocity is higher, they tend to split into pairs of waves moving in opposite directions. The length of the wave appears to be a crucial to this behavior: shorter waves are more likely to transition to rotating waves, while longer waves are stable up to a critical length at which local excitation in the tail of the wave becomes stronger than the inhibition from the front, resulting in the formation of a secondary front moving in the opposite direction. This critical length is approximately twice the radius of a stable bump solution.

### 3.3. Segmentation of Parameter Space Based on Behavior of Propagating Waves

**Figure 6:**
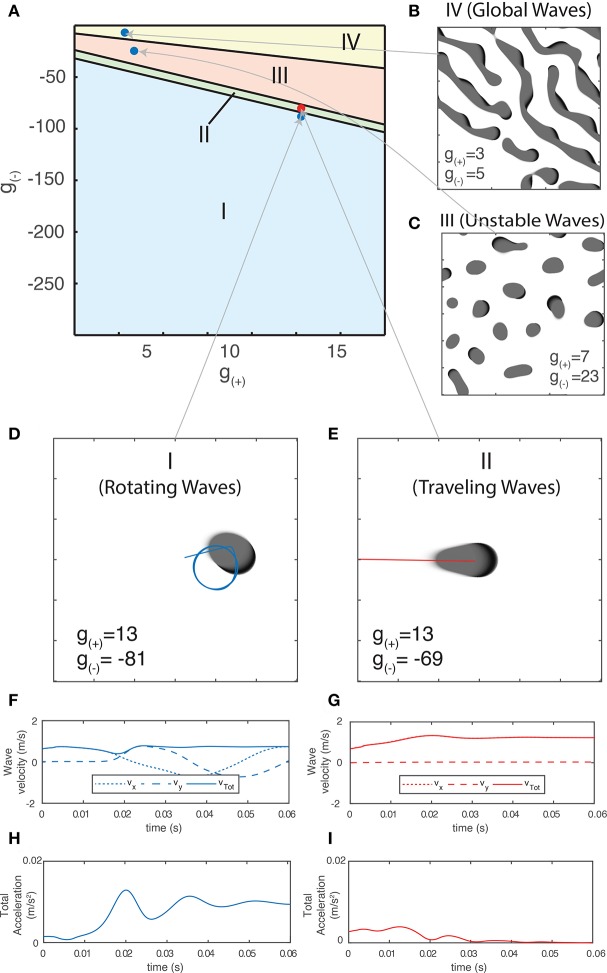
**(A)** Characterization of the parameter space based on the dominant type of pattern seen in numeric simulations. **(B–E)** Snapshot images of the neural field at various parameter values. Center of mass trajectories (*x*_*t*_, *y*_*t*_) for isolated waves are overlaid in **(D,E)**. **(F,G)** Velocity time series *v*_*x*_, *v*_*y*_, *v*_*tot*_ of the trajectories of individual waves as plotted in **(D–E)**, found by differentiating *x*_*t*_, *y*_*t*_ numerically. **(H,I)** Total acceleration time series *a*_*tot*_ found by differentiating *v*_*tot*_ numerically. The total velocity and acceleration of individual waves are both constant and positive for rotating waves while stable traveling waves have *v*_*tot*_ > 0 and *a*_*tot*_ = 0.

Using the method outlined in section 3.2.1.1, we are now able to generate a traveling wave for any set of parameters. For many values of *g*_±_, we find that the traveling waves are unstable and either periodically divide or transition to a rotating wave. We use this property of how individual waves behave in order to separate the parameter space into four distinct regions based on the dominant form of activity, as shown in Figure 6A. Region I - Rotating waves: Asymmetrical patterns which move in a circular orbit about a fixed point as in Figure 6D. Region II - Traveling waves: Symmetric patterns which move in linear trajectory with constant speed and length as in Figure 6E. Region III - Unstable waves: Traveling waves whose length and speed keeps increasing until they split into two waves propagating in opposite directions as seen in Figure 5A. Due to this, over time the field becomes filled with patterns that are spatially confined to a small region of the field as shown in Figure 6C. Region IV - Global waves: Extended, slowly-moving patterns with multiple “fronts” or bands of activation that fill the neural field, shown in Figure 6B.

### 3.4. Characterization of Stability Using Direct Numerical Simulations

The non-linearity present in the neural field equations (Equation 4) makes it difficult to determine the stability of the traveling wave solutions analytically, so we use a computational approach to produce a phase portrait and identify its equilibria, similar to methods that have been applied to experimental data from unstable dynamical systems (Wiebe and Virgin, 2016). For our state variables, we track the trajectories of the waves using their center of mass coordinates, which we then use to calculate their velocity and acceleration over time. The instantaneous values of these variables can be used to distinguish between the different types of patterns. The variables also show a smooth transition between them in phase space. We outline these variables and how they describe different pattern types below.

The center-of-mass coordinates $\vec{\rho }\left(t_{k}\right)$ of the wave *S* at each time step *t*_*k*_, are found using:

(14) $$\begin{array}{r} \underset{\text{r}\in S}{\sum }\left(f\left(\text{r},t_{k}\right)-\vec{\rho }\left(t_{k}\right)\right)=0 \end{array}$$

The wave velocities are numerically calculated using the finite difference method: **v**(*t*_*k*_) = 0.5 × [**r**(*t*_*k*+1_) − **r**(*t*_*k*−1_)]/Δ*t*. Smoothing is then applied using a 10-point moving average. Acceleration **a**(*t*_*k*_) is calculated similarly from the velocity. The norm of the velocity and acceleration vectors is taken to calculate their total velocity *v*(*t*_*k*_) = |**r**(*t*_*k*_)| and total acceleration *a*(*t*_*k*_) = |**a**(*t*_*k*_)|.

Different pattern types have unique trajectories which give rise to varying velocity and acceleration profiles. For a single wave, the velocity in a fixed direction (e.g., along the *x*-axis) along with total velocity and acceleration are the most useful quantities for classifying them numerically. In Figure 6D, we show the center-of-mass trajectory of a rotating wave, with the figures below showing its velocity and acceleration. Isolated rotating waves have constant positive total acceleration and total velocity, i.e., *a*_tot_ > 0 and *v*_tot_ > 0. In Figure 6E, we repeat this for traveling waves, which have constant velocity in the direction of propagation. Hence, they have *a*_tot_ = 0 and *v*_tot_ > 0.

**Figure 7:**
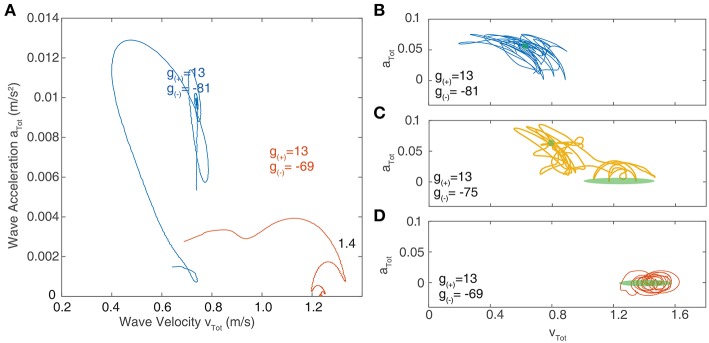
**(A)** Velocity-acceleration phase portrait showing the trajectories of traveling waves after a single perturbation. The different paths correspond to different levels of inhibition near the critical point of transition between region I and II as shown in Figure 6A. Stationary bumps are represented by a single point at the origin; rotating waves have non-zero acceleration, hence they lie in the positive region of the y-axis; stable traveling waves have zero acceleration and lie along the x-axis. **(B–D)** Phase portraits of traveling waves with continuous noisy inputs. The green shaded regions indicate the location of stable solutions in the absence of noise. **(B)** At parameter values lying in region I (as outlined in Figure 6), the trajectory of waves in phase space oscillates around the region corresponding to stable rotating waves (shown in green). **(C)** At parameter values in between region I and II, the trajectory of waves in phase space oscillates between the regions corresponding to stable traveling waves and rotating waves. **(D)** At parameter values in region II, the trajectory only oscillates around the region corresponding to stable traveling waves.

We now focus on the region near the boundary separating rotating waves and traveling waves to explore their stability and the nature of the transition between these two regimes. Figures 6D,E show a snapshot of the neural field at two values of *g*_(±)_ around the transition region. Figures 6F–I show the corresponding wave velocity and acceleration. We see that after a transient period approximately 50 ms, both waves stabilize with *v*_tot_ > 0 and *a*_tot_ > 0 for the rotating wave and *v*_tot_ > 0 and *a*_tot_ = 0 for the traveling wave. In Figure 7A, we show the trajectories of the waves shown in Figures 6D,E on a phase diagram with *v*_tot_ and *a*_tot_ on the *x* and *y* axes, respectively. To better explore the dynamical nature of the wave behavior, we initialize the neural field with a traveling wave and apply a single perturbation in the form of uniform Gaussian noise. From their initial state, we see a smooth transition toward their final state with several oscillations before they settle into a steady state.

At the critical point of transition between the two regimes, both types of waves coexist as bistable equilibria; rotating waves and traveling waves can be observed and a sufficiently strong perturbation can induce a transition from one to the other. To demonstrate this, we apply continuous perturbations to the traveling waves at each integration step, represented as an added noise term μ to Equation (4):

$$\begin{array}{l} \frac{\partial f}{\partial t}=-f+\left(1-f-h\right)H\left(u-\kappa \right)+\mu \\ \frac{\partial h}{\partial t}=-ph+f-\mu \end{array}$$

Where μ = *f* × ξ, and ξ is Gaussian white noise with zero mean and variance $\sigma_{\xi }^{2}$. Modeling noise in this way allows us to ignore the effect of noise on other aspects of the neural field, such as modifying the radius of bumps or spontaneously generating new patterns in other regions of the field.

Figures 7B–D show the resulting trajectories in phase space for the same parameter values as in Figure 7A as well as an additional parameter value that lies between these. For the noise term, we use σ_ξ_ = 0.2. The region of phase space corresponding to the stable value in the absence of noise highlighted in green. For parameter values drawn from the “Rotating waves” region of Figure 6A, the trajectory oscillates around the equilibrium corresponding to a stable rotating wave (Figure 7B). For a wave in the “Traveling Wave” region of parameter space (Figure 7D), it oscillates about the equilibria for traveling waves. If we adjust the parameters to lie in between these two regimes (Figure 7C), then the trajectory in phase space passes through both equilibria, demonstrating the bistability of the system.

**Figure 8:**
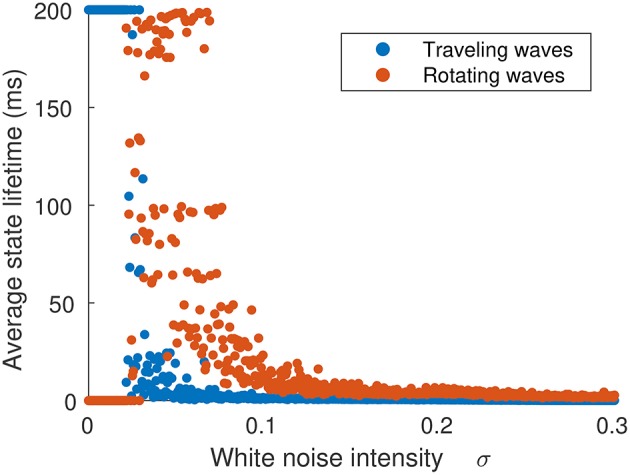
Average time spent in either traveling or rotating wave state in terms of the strength of noise inputs. The initial state was a traveling wave. When noise is weak (σ_ξ_ < 0.026), no transitions occur. As noise is increased (σ_ξ_ > 0.026), traveling waves transition to rotating waves, which have a longer lifetime. When noise is very strong (σ_ξ_ > 0.06), the waves do not settle into single configuration and their trajectories explore the full state space.

The probability of a transition occurring is dependent on σ_ξ_, as shown in Figure 8, which shows the average lifetime of each state when the neural field is simulated for 60 ms for the same parameter value as Figure 7C. At low levels of σ_ξ_, traveling waves do not transition to rotating waves, but as σ_ξ_ is increased above 0.26, transitions happen with increasing frequency, although rotating waves appear more often than traveling waves.

### 3.5. Characterization of Collective Dynamics of Interacting Patterns

**Figure 9:**
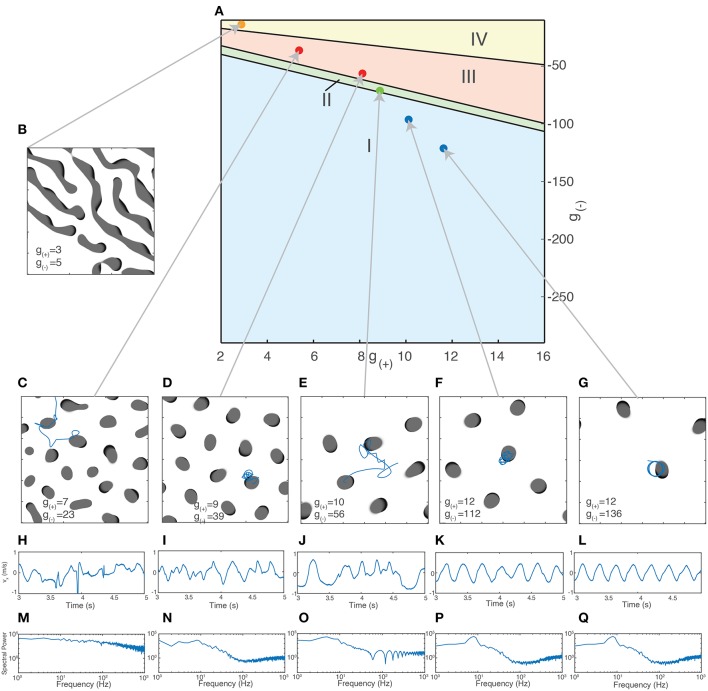
Different types of collective behavior emerge when the excitatory/inhibitory strength is varied. **(A)** Dots indicate parameter values used for simulations of collective activity. Values were chosen so that the size of patterns remained relatively constant (as measured by the radius of bump solutions given by Equation 7). **(B–G)** Snapshots of neural field activity at various parameter values show the change from global waves and Turing patterns **(B)** to interacting bumps of irregular sizes **(C)**, bumps of similar size aligned to a grid **(D)**, interacting traveling waves **(E)** and isolated rotating waves **(F,G)**. **(H–L)** Velocity time series *v*_*x*_ of a single wave along a fixed direction shows the changing characteristics of wave movement as parameter values are changed, from irregular movement driven by frequent collisions **(H–J)** to regular oscillations **(F,G)**. **(M–Q)** Periodogram (Fourier transform) of the time series *v*_*x*_ shows a transition from a relatively flat power spectrum **(M)** to one with a clear peak corresponding to the frequency of the rotating waves' oscillation **(K,L)**.

In addition to the behavior of isolated patterns, we study the impact of varying excitatory and inhibitory strength on the collective behavior of multiple interacting patterns and quantify these differences using the statistical properties of their motion. In order to reduce the variance that may arise due to the spatial discretization, field size, and periodic boundary conditions, we fix the size of the neural field and the size of patterns while varying the excitation and inhibition. As the value *g*_±_ influences the size of the patterns, as shown earlier in Figure 3A, we simulate only a subset of all the possible *g*_±_ values. This subset was found by calculating the upper bump radius for the *g*_±_ values obtained using the Monte Carlo method using Equation (7) and then filtering only those whose bump radius corresponds to around (3.00±0.01) mm, consistent with the size of experimentally observed waves in the cortex (Han et al., 2008). These values lie near a curved region in the parameter space, shown in Figure 9. As the size of rotating and traveling waves is very close to that of radial bumps, this ensures that the pattern size remains constant over the range of parameters.

For the initial state of the simulation, we fix the number of patterns present. If there are too few patterns, they interact rarely and the resulting behavior is similar to that of isolated patterns. There is also a maximum number of patterns that can exist in the neural field at the same time, which depends on the size of the patterns and the strength of the long-range inhibition. For patterns of length 6 mm with a square grid length of 60 mm, this limit is in the range of 9–14 patterns. At this limit, the patterns will be spatially confined to a grid due to repulsive interactions arising from long-range inhibition. In order to observe and quantify the changing nature of the interactions between the patterns, we simulate a constant, fixed amount; we chose 7 so that interactions between patterns would occur regularly but they would initially not be spatially confined to a grid.

We initialize the patterns as traveling waves with random orientations and positions. After simulating for sufficiently long time to eliminate transient activity (4s), we inspect the activity visually and calculate their trajectories through their center-of-mass coordinates. Figures 9C–G shows snapshots of the neural field and the motion of the patterns as *g*_(±)_ is varied. Shown in Figures 9F,G is the behavior at high levels of inhibition, with the spatiotemporal patterns following periodic, isolated rotating trajectories. As inhibition is weakened, the trajectories become more varied and dynamic due to increased interactions between the patterns and the increasing stability of traveling waves (Figure 9E). As inhibition is weakened further, past the transition point where traveling waves become unstable (Figures 9C,D), the waves multiply and divide, filling the neural field and becoming confined to isolated positions within a hexagonal grid. As inhibition is weakened further, near the region where global waves dominate (Figure 9C), the patterns begin to show much more diversity in their size and appearance and are no longer confined to a grid. In the region where global waves dominate (Figure 9B), we no longer see isolated bump-like patterns but long, slowly-moving wave fronts.

To quantify these differences, we use the trajectories of the waves over time for time series analysis. We calculate the center-of-mass coordinates and velocities as previously described using Equation (15). These are shown in Figures 9H–L. We apply a Hanning window to the velocity data then take a Discrete Fourier Transform (DFT), after a sufficient transient period (3s). We calculate the *N* normalized coefficients of the power spectrum *S*_*i*_ and use it to produce the periodograms in Figures 9M–Q. The regular oscillations seen in the states in Figures 9F,G are reflected in the peak in their respective periodograms around 9 Hz. This disappears as inhibition is reduced and the motion becomes more chaotic, as shown in Figure 9E. The other components of the spectrum, however, remain fairly consistent until the onset of chaotic behavior as seen in Figure 9C. For global wave activity as in Figure 9B, we did not calculate the wave velocity as the large spatial extent of the wave fronts made the center of mass coordinates a less useful metric for quantifying the wave behavior. To quantify the changes in spectral profile and the velocity time series, we use three different statistics: The degrees of freedom (DoF) of the power spectrum, the approximate entropy (ApEn) of the wave velocity time series, and the Detrended Fluctuation Analysis (DFA) exponent of the wave velocity. The DoF statistic measures the uniformity of the power spectrum (Vaillancourt and Newell, 2003) and is defined as:

(15) $$\begin{array}{r} \text{DoF}=\frac{N{\left(\sum_{i}^{N}S_{i}\right)}^{2}}{\sum_{i}^{N}S_{i}^{2}} \end{array}$$

**Figure 10:**
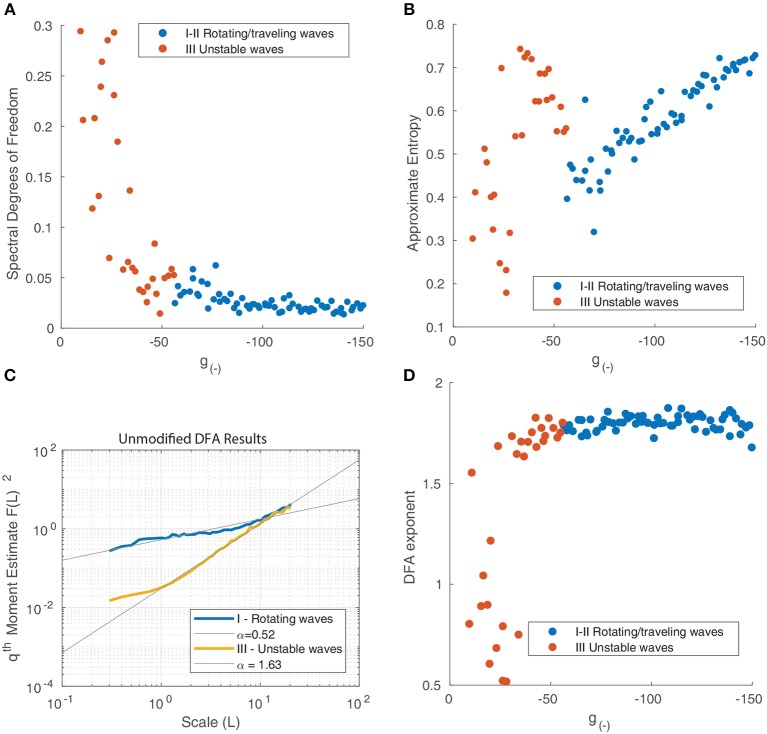
**(A)** Spectral degrees of freedom parameter vs. the inhibitory strength for the motion of waves in Figure 9. This parameter quantifies the uniformity of the spectral distribution and shows that the distribution is uniform at low levels of inhibition and non-uniform when inhibition is high, reflecting the transition from random motion to periodic oscillation. **(B)** Approximate Entropy vs. the inhibitory strength. This quantifies the degree of irregularity in the motion of the waves which peaks for unstable waves, around the point of transition between similarly-shaped waves aligned to a grid (Figure 9D) and waves of varying size (Figure 9C). **(C)** Detrended fluctuation analysis of the time series of wave velocity for two parameter values, the exponent α is represented by the gradient of the trend line. A value of 1.5 corresponds to a Brownian motion process (random walk), while a value of 1 corresponds to 1/*f* noise; a value of 1/2 is associated with white noise. **(D)** DFA exponents α of the wave velocity for various parameter values. For most parameter values, the DFA lies in the range 1.5–2, consistent with a non-stationary process that can be modeled as fractional Brownian motion. At decreasing levels of inhibition, the motion becomes increasingly random, reflect by the DFA exponent decreasing and reaching a minimum of 0.5.

For a uniform power spectrum (white noise), the DoF is equal to 1, whereas for a delta-function shaped power spectrum, it approaches 0. The DoF statistic increases as inhibition is reduced (Figure 10A), indicating that the power spectrum of the motion of the waves becomes more uniform as the periodic components associated with the motion of isolated rotating waves disappear and the motion becomes more random.

The ApEn quantifies the amount of regularity and unpredictability of the fluctuations in a time series. The ApEn returns a value between 0 and 2 which reflects the predictability of future values of a time series based on previous values. To calculate ApEn, we followed the method outlined in Pincus (1991). As shown in Figure 10B, the Approximate Entropy peaks around the transition between similarly-shaped waves aligned to a grid and waves of varying size, indicating that the motion at these parameter values is the most chaotic.

We next perform a Detrended Fluctuation Analysis (DFA) on the time series *v*_*x*_(*t*_*k*_) to analyze the scale-free decay of temporal (auto)correlations, also known as long-range temporal correlations (LRTC). The DFA measures the power-law scaling of the root-mean-square fluctuation of the integrated and linearly detrended signals as a function of time window size. It is calculated as follows (Chen et al., 2002):

1. The velocity time series *v*_*x*_(*t*_*k*_) is integrated and the mean subtracted using $y\left(k\right)=\overset{k}{\underset{i=1}{\sum }}\left[v_{x}\left(t_{i}\right)-\langle v_{x}\left(t_{i}\right)\rangle \right]$.
2. The integrated signal is divided into boxes of equal length *n*.
3. In each box we fit a linear trend to the time series which we denoted *y*_*n*_(*k*).
4. The integrated signal is detrended by subtracting the local trend *y*_*n*_.
5. For a given box size *n*, the root-mean-square (rms) fluctuation for this signal is calculated:
  (16) $$\begin{array}{r} F_{DFA}\left(n\right)=\sqrt{\frac{1}{N_{max}}\overset{N_{max}}{\underset{k=1}{\sum }}{\left[y\left(k\right)-y_{n}\left(k\right)\right]}^{2}}. \end{array}$$
6. The above calculation is repeated for a broad range of *n* to give a relationship between *F*_*DFA*_(*n*) and n.
7. A power-law exponent α is fitted to the fluctuation function $F_{DFA}\left(n\right)n^{\alpha }$.

The DFA exponent α is the slope of the fluctuation function and can be related to the power-law scaling exponent of the autocorrelation function. For a particular time series, a DFA exponent of 0.5 indicates a signal that is without autocorrelations (white noise), whereas a DFA exponent between 0.5 and 1.0 indicates the presence of scale-free temporal correlations (autocorrelations) (Linkenkaer-Hansen et al., 2001; Chen et al., 2002; Gao et al., 2006). A value of 1 is associated with 1/*f* noise (pink noise); a value above 1 is associated with an unbounded, non-stationary signal, with a value near 1.5 associated with Brownian noise. We follow the method outlined in Linkenkaer-Hansen et al. (2001) to calculate the DFA exponents. The results for two distinct states - rotating waves and traveling waves - are presented in Figure 10C to highlight how this metric quantifies the changing behavior of the neural field.

We find that most states where characterized by an exponent slightly above 1.5, however this decreased suddenly for unstable states (Figure 10D), indicating that the motion becomes increasingly random and uncorrelated. The decrease in α as inhibition decreases can be explained by considering the impact of decreasing inhibition on the stability of patterns. This decrease in stability allows for a much wider range of accessible states and interactions. At any one point in time *t*_*k*_, a diverse range of pattern shapes and sizes can be seen, as seen in Figure 6E.

### 3.6. Characterization of Long-Range Spatiotemporal Correlations Using Spectral Analysis

We then study the spatiotemporal spectrum of the neural field activity. To do so, we simulate the neural field for 4 s (3,000 time steps) in order to eliminate transient states and apply a Discrete Fourier Transform (DFT) to the final portion of the neural field output using the FFTW library (Frigo and Johnson, 2005). As before, our choice of initial condition is 7 randomly distributed traveling waves. This configuration allows us to observe the collective dynamics of patterns in the regime where traveling waves interact regularly but still have some freedom to move (i.e., not confined to a grid due to their repulsive interactions). As we have periodic boundary conditions in the spatial dimensions, we only apply a Hann (Hanning) window function along the temporal dimension, as outlined in Oppenheim et al. (1999). We take the absolute value of the complex output and, assuming rotational invariance, we average the spatial dimensions radially similar to Dong and Atick (1995). As a result, the final spectrum has one spatial and one temporal dimension.

**Figure 11:**
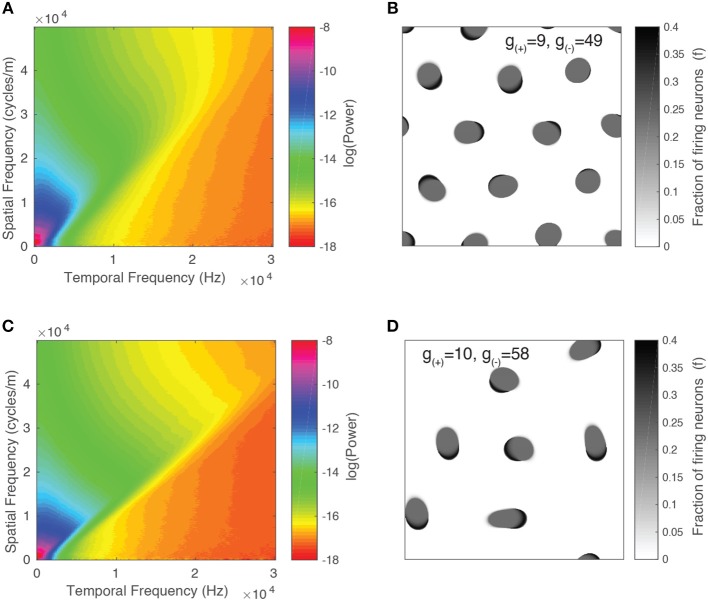
Spatiotemporal spectra for two parameter values alongside a snapshot of the neural field activity. **(A,B)** Unstable waves confined to grid. Most of the spectral power is concentrated at lower frequencies and falls off at higher frequencies. **(C,D)** Stable traveling waves. Spectral power is also concentrated at lower frequencies, however, the power drops off rapidly once it crosses a line of constant *f*/ω, which is consistent with the maximal velocity of the waves.

We present the spectrum for two parameter values in Figures 11A,C. Alongside each spectrum is a snapshot image of the corresponding neural field (Figures 11B,D). The parameter values chosen lie on either side of the transition from stable to unstable traveling waves. In both spectra, the power peaks at the origin and is concentrated along lines of constant spatial/temporal frequency ratios, i.e., $\frac{\omega }{f}$. This can be explained by considering the Fourier transform of a single object moving with velocity *v*. As demonstrated by Watson and Ahumada (1985), the resulting spectrum is a distortion of the power spectrum of a stationary object which lies along a line described by:

(17) $$\begin{array}{r} vf+\omega =0. \end{array}$$

For multiple objects moving in two dimensions with a range of velocities, the resulting spectrum reflects the distribution of velocities among the objects (Dong and Atick, 1995). Following the approach of Rivait and Langer (2007), we explore this by calculating the total power along lines of constant velocity i.e., $v=\frac{\omega }{f}$. That is, for a range of velocity values *v* and temporal frequencies ω, we linearly interpolate the power spectrum Ŝ above and below the line $f=\frac{\omega }{v}$ and sum the resulting values:

(18) $$\begin{array}{r} {Ŝ}_{tot}=\underset{\omega }{\sum }Ŝ\left(v,\omega \right) \end{array}$$

**Figure 12:**
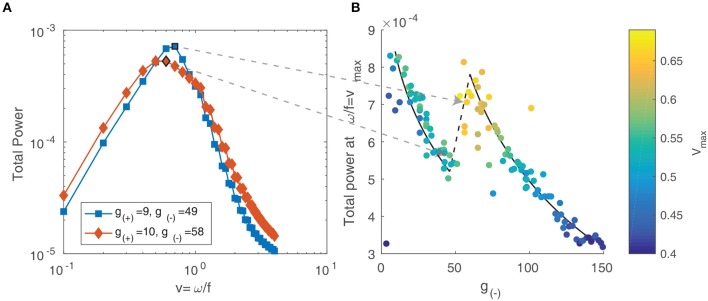
**(A)** Total spectral power vs. velocity, expressed as the ratio of temporal and spatial frequency. **(B)** The peak value of velocity-power curves as in **(A)** vs. the parameter *g*_(−)_. The color scale represents the velocity value of the peak, which increases near the transition between stable and unstable traveling waves.

We show the result in Figure 12A and observe that for both parameter values, the power peaks at a particular value of *v* and decays away from this value. The peak value and the corresponding *v* value varies between different parameter values, reflecting the changing distribution of velocities due to interactions between patterns and their maximum velocity. We characterize this by simulating the neural field for the same range of parameter values as in the previous section and calculating their spectra. In Figure 12B, we show the peak power values as a function of the parameter *g*_(−)_, with the color scale showing the corresponding velocity. We observe that the power and velocity peaks near the previously identified transition point between stable and unstable traveling waves. This reflects that near this point, waves in the neural field are most mobile; their velocity is maximized and they are able to propagate with fewer interactions with other waves than when the neural field is filled with patterns.

## 4. Discussion

In this study we have investigated the neural mechanism of the formation of complex propagating wave dynamics in a 2D neural field model. We have found that, as excitation increases or inhibition decreases, the dominant form of activity transitions from stationary bumps to rotating waves, to traveling waves, to unstable waves, and finally to global waves. These transitions can be used to define distinct regions in the parameter space. Using linear stability analysis, we verified these results by showing that an increase in excitation and a decrease in inhibition both resulted in the system moving further away from the region where stationary bumps are stable. At the transition between these regions, we have observed bi-stability of different kinds of spatio-temporal patterns: under isolated conditions, each pattern is stable, but perturbations can induce transitions between the states. Furthermore, continuous switching between stable states is possible in the presence of constant external noise.

This provides insight into the underlying mechanisms of the behavior of spatiotemporal patterns seen in experiments, such as switching between different types of activity patterns in local cortical circuits. This type of switching has been observed between spiral, plane, ring and irregular waves in disinhibited mammalian cortex (Huang et al., 2004), as well as a range of source/sink, spiral, saddle, and other patterns in primate and mouse cortex (Townsend et al., 2015). The exact mechanisms behind these changes are still unclear, however our results show that two possible factors are the effects of external noise and short-term modulations in the level of excitation or inhibition which could shift the phase landscape and induce a transition between states. In our neural field model, the level of excitation and inhibition is given by the weight function (Equation 3), which expresses the synaptic inputs to a point in the field based on activity in the rest of the field. This is affected by the synaptic connectivity, as well as the action of neurotransmitters, external inputs, and neural “noise.” There are several mechanisms by which this could be modified, including synaptic plasticity (Sussillo and Abbott, 2009; Vogels et al., 2012), the influence of endogenous substances and drugs (Behrends and ten Bruggencate, 2017), and changes in external inputs due to shifts in behavioral states (Taub et al., 2013; Zhou et al., 2014). These various mechanisms operate across a range of timescales that vary over several orders of magnitude, which could help account for the diverse range of spatiotemporal phenomena, such as the stable, rhythmic waves seen in human primary motor cortex (Takahashi et al., 2011). Future studies could experimentally verify these mechanisms and compare to the output of our model, similar to the work of Golomb and Amitai (1997) and Richardson et al. (2005).

In addition to single patterns, we have also studied the collective dynamics of multiple interacting patterns and the influence of the level of excitation/inhibition. Analyzing the statistical properties of the collective motion reveals a range of collective propagation dynamics, including interacting propagating waves with Brownian motion, random motion within patterns spatially confined to a grid, irregular patterns with unstable dynamics and slowly moving global waves. The quality of the motion, as revealed by statistical methods such as DFA, is influenced by the level of excitation and inhibition and shows dramatic changes at particular transitions in state space, such as when traveling waves become unstable. Long-range spatiotemporal correlations are also revealed by the spatial and temporal power spectrum of the neural field activity. These spatiotemporal correlations arise from the unique distribution of traveling waves in space and their motion within the neural field. Consistent with our analysis using other methods, we observe a shift in the spectrum as the activity changes due to changing levels of excitation and inhibition. This is linked to a change in the distribution of velocities among patterns in the neural field. Our results thus advance existing studies of neural fields which have mainly focused on single waves or multiple waves with regular dynamics (Kilpatrick and Bressloff, 2010; Bressloff and Kilpatrick, 2011; Meijer and Coombes, 2014).

The presence of balanced excitation and inhibition in cortical activity has been well established experimentally *in vivo* (Haider, 2006; Okun and Lampl, 2008; Xue et al., 2014) and it is believed to be essential for normal brain function. Our results show that changing levels of excitation and inhibition could be a mechanism for regulating the dynamics of spatiotemporal activity in neural systems.

## Data Availability

The raw data supporting the conclusions of this manuscript will be made available by the authors, without undue reservation, to any qualified researcher.

## Author Contributions

This study was co-designed by DN and PG. DN performed the analysis and wrote the drafts of the manuscript with PG.

### Conflict of Interest Statement

The authors declare that the research was conducted in the absence of any commercial or financial relationships that could be construed as a potential conflict of interest.
